# Genetic monogamy despite frequent extrapair copulations in “strictly monogamous” wild jackdaws

**DOI:** 10.1093/beheco/arz185

**Published:** 2019-11-22

**Authors:** Lisa F Gill, Jaap van Schaik, Auguste M P von Bayern, Manfred L Gahr

**Affiliations:** 1 Department of Behavioural Neurobiology, Max Planck Institute for Ornithology, Eberhard-Gwinner-Strasse, Seewiesen, Germany; 2 Department of Applied Zoology and Nature Conservation, University of Greifswald, Greifswald, Germany; 3 Department of Biology II, Ludwig-Maximilians-Universität München, Planegg-Martinsried, Germany

**Keywords:** bio-logging, breeding success, copulation calls, corvids, extrapair copulations, jackdaws (*Corvus monedula*), microphone backpacks, monogamy, sexual behavior, sexual conflict

## Abstract

“Monogamy” refers to different components of pair exclusiveness: the social pair, sexual partners, and the genetic outcome of sexual encounters. Avian monogamy is usually defined socially or genetically, whereas quantifications of sexual behavior remain scarce. Jackdaws (*Corvus monedula*) are considered a rare example of strict monogamy in songbirds, with lifelong pair bonds and little genetic evidence for extrapair (EP) offspring. Yet jackdaw copulations, although accompanied by loud copulation calls, are rarely observed because they occur visually concealed inside nest cavities. Using full-day nest-box video surveillance and on-bird acoustic bio-logging, we directly observed jackdaw sexual behavior and compared it to the corresponding genetic outcome obtained via molecular parentage analysis. In the video-observed nests, we found genetic monogamy but frequently detected forced EP sexual behavior, accompanied by characteristic male copulation calls. We, thus, challenge the long-held notion of strict jackdaw monogamy at the sexual level. Our data suggest that male mate guarding and frequent intrapair copulations during the female fertile phase, as well as the forced nature of the copulations, could explain the absence of EP offspring. Because EP copulation behavior appeared to be costly for both sexes, we suggest that immediate fitness benefits are an unlikely explanation for its prevalence. Instead, sexual conflict and dominance effects could interact to shape the spatiotemporal pattern of EP sexual behavior in this species. Our results call for larger-scale investigations of jackdaw sexual behavior and parentage and highlight the importance of combining social, sexual, and genetic data sets for a more complete understanding of mating systems.

## Teaser

Monogamy is defined at the social, sexual, and genetic levels—which do not necessarily match. Based on genetic findings, jackdaws are considered a rare example of avian monogamy. In a colony of wild jackdaws, we found that extrapair (EP) copulations occurred frequently but without yielding EP offspring. This behavior was seemingly male driven and forced and prevented by mate guarding during female fertility. Our results highlight the importance of combining genetics and behavior to better understand mating systems.

## INTRODUCTION

Before the rise of molecular tools, it was commonly agreed that birds were exceptional because, in most species, individuals choose one exclusive mating partner for life (Lack 1940). With genetic evidence for extrapair (EP) offspring accumulating across taxa, even for textbook monogamous species (Huyvaert et al. 2000; Jouventin et al. 2006), absolute monogamy—exhibited at the social, sexual, and genetic level—is now considered an exception in birds (Griffith et al. 2002). Today, most studies focus mainly on the genetic component of monogamy because it is strongly linked with sexual selection and conflict (Westneat and Stewart 2003; Kempenaers and Schlicht 2010; Chaine et al. 2015). Yet, just as the observed social behavior often turned out to be inconsistent with the corresponding genetic evidence, avian sexual behavior may not be accurately reflected by its genetic outcome (Emlen and Wrege 1986; Kempenaers and Schlicht 2010) because diverse strategies have evolved that may alter fertilization rates, for example, behavioral and anatomical adaptations or postcopulatory sperm selection (Hasselquist 2001; Griffith et al. 2002; Westneat and Stewart 2003; Birkhead 2010). For instance, male mate guarding and frequent intrapair (IP) copulations have been shown to reduce the likelihood of EP fertilizations in birds (Møller and Birkhead 1991; Kokko 2005; Harts et al. 2016). Also, the timing of copulations, as well as hatching and fledging success, may differ systematically between IP and EP contexts (Griffith et al. 2002; Krist et al. 2005; Vedder et al. 2012; Safari and Goymann 2018), which in turn may lead to inconsistent behavioral and genetic findings.

However, studies directly comparing the levels of social, sexual, and genetic aspects of avian monogamy are rare. Obtaining systematic recordings of sexual behavior is challenging in birds because copulations are usually short (Birkhead et al. 1987) and may occur unpredictably and cryptically—especially in EP contexts, which might result in physical fights or reduced parental care when detected by conspecifics (Hauser 1998; Valera 2003; Westneat and Stewart 2003; Kempenaers and Schlicht 2010). Thus, because quantifications of avian sexual behavior are scarce, the success rates of IP and EP copulation behavior remain largely unknown, leaving the factors that shape them unexplored.

Jackdaws (*Corvus monedula*) are group-living corvids that form lifelong pair bonds (Lorenz 1931; Roell 1978; Dwenger 1989; Cramp and Perrins 1994; Liebers and Peter 1998; Henderson et al. 2000) and serve a rare example for “strict monogamy” (Henderson et al. 2000) in birds. However, because jackdaw copulations occur visually concealed inside nest cavities, direct observations of sexual behavior are often lacking (Dwenger 1989; Cramp and Perrins 1994; Liebers and Peter 1998). Thus, previous investigations of jackdaw monogamy were based solely on small-scale genetic data sets and general observations but not on sexual behavior (Liebers and Peter 1998; Henderson et al. 2000). Thus, it remains unclear whether negative evidence for EP fertilizations reported by previous studies was caused by the strict absence of EP copulations or rather by failed EP fertilizations or missing DNA samples of EP chicks.

During otherwise concealed copulations, jackdaws produce loud “räääh-räääh-räääh” calls (Dwenger 1989). These can be heard from a distance and elicit strong behavioral responses in the colony (increased agitation, nest-site inspections; personal observations), but it is unknown which sex produces these calls and what their function is (Dwenger 1989; Cramp and Perrins 1994). In other animal species, “copulation calls,” that is, characteristic vocalizations uttered during copulation, have been studied in wider evolutionary contexts because they may bear costs and benefits to the signaling or receiving individuals. On the one hand, being noisy and unvigilant during copulation may increase predation risk (Siemers et al. 2012) and exacerbate intraspecific conflict (Townsend and Zuberbuhler 2009). On the other hand, copulation calls may play an important role in mate attraction (Hauser 1998; Anoop and Yorzinski 2013; Løvlie et al. 2014), stimulation, and synchronization of sexual partners (Brockway 1965; Lehrman and Friedman 1969; Hauser 1998), as well as in postcopulatory sexual selection (Birkhead 2010). However, although a plethora of studies focused on vocal behavior associated with reproduction, comparably little is known about vocalizations during copulation, especially in birds (Hauser 1998; Bradbury and Vehrencamp 2011; Schlicht and Kempenaers 2016). Until recently, identifying the sound-producing individuals was challenging when visual confirmation was unavailable or when animals vocalized in close proximity to each other as during copulation. Today, miniaturized onboard microphones allow recording individual-level vocalizations in freely behaving birds (Gill et al. 2016) and contribute information on the behavioral and social contexts in which these vocalizations occur (Stowell et al. 2017; Greif and Yovel 2019).

The first aim of our study was to find out whether the previous findings of rare songbird “strict monogamy” (Henderson et al. 2000) could be reproduced in a wild colony of jackdaws if not only the genetic aspect was investigated in detail but also the social, sexual, and genetic level of monogamy were exhaustively quantified and directly compared. Next, we aimed to explore reasons for potential mismatches between levels in the degree of monogamy, such as systematic differences in EP offspring survival (e.g., fertilization, hatching, or fledging rates; Hasselquist 2001; Griffith et al. 2002; Birkhead 2010; Kempenaers and Schlicht 2010; Ferree et al. 2010), sexual behavior (e.g., solicited versus forced copulation attempts; Dunn et al. 1999), or sampling bias. Further, in many bird species, mate guarding and increased copulation frequencies may not only prevent EP fertilizations but also bear costs, such as increased predation risk, energy demands, or reduced time allocated to foraging or seeking EP sexual opportunities (Møller and Birkhead 1991; Kokko 2005; Harts et al. 2016). According to the paternity assurance hypothesis, males would, thus, limit their engagement in such behaviors to their female’s fertile phase (Hunter et al. 1992; Villarroel et al. 1998; Harts et al. 2016). We were, thus, interested in whether IP copulation rates and the time spent by a male or female in the nest box alone or with the partner would change with female fertility status and affect EP fertilization rates. Lastly, EP copulations may yield direct material benefits or increase male and female fitness via EP fertilizations and are actively sought out by both sexes in many bird species (Kempenaers and Schlicht 2010). Because previous studies found no evidence for high rates of EP fertilizations in jackdaws (Liebers and Peter 1998; Henderson et al. 2000) and our initial observations suggested that EP sexual behavior was forced and associated with potentially costly fights, we aimed to explore underlying causes for EP sexual behavior in this species.

Here, we used a combination of 1) observations and nest checks to monitor pairs and breeding success, 2) nest-box cameras to quantify nest-box occupancy and sexual behavior, 3) microphone backpacks for individual-level vocal and nonvocal sound recordings, and 4) molecular parentage analysis of adults (blood samples) and hatchlings (buccal swabs) to track parentage in a colony of wild jackdaws.

## METHODS

The sociosexual behavior and reproductive success of a colony of wild jackdaws was investigated during multiple breeding seasons (main study period: 2017) using field observations (2013–2017), nest checks and audio–video nest-box surveillance (2013–2015, 2017), acoustic bio-logging and radio-telemetry (2014, 2015), and molecular parentage analysis (2017). Procedures were in accordance with the European directives for the protection of animals used for scientific purposes (2010/63/EU) and were granted approval by the Government of Upper Bavaria. The established colony contained around 20 pairs nesting in wooden nest boxes (ca. 80% of the colony), chimneys, and crevices in and around a large building in a village close to Starnberg, Bavaria, Germany. Sixteen nest boxes, accessible from inside the building, were equipped with small trap doors and large apertures before the breeding season for capturing and taking out the birds. Eight of these were video observed for behavioral quantifications in 2017 (see below).

### Nest checks and female fertility

During most of the breeding period, nest checks were carried out every 2–3 days during midday when the adult jackdaws were least likely to be around. During egg laying and hatching, nest checks were performed daily, in the mornings, to allow individual marking of eggs and chicks and for individual DNA sampling (see below).

Eggs were numbered with a felt-tipped pen and chicks were individually marked via nail polish applied to their toenails. Chicks with good fledging prospects obtained a numbered aluminum ring (minimum age: 21 days). Eggs and young chicks were weighed using a digital balance (to the next 0.01 g) and older chicks via a spring balance (see below). We checked by hand whether eggs were cold or warm to approximate the onset of incubation.

We recorded nest-building status (presence and type of nest material), the presence of eggs, and the development of the corresponding chicks until fledging to determine a resident pair’s (*n*_2017_ = 16) breeding stages (prelaying: no activity, nest building; egg laying; incubation; chick rearing: hatchlings, young chicks, older chicks; all chicks gone: dead or fledged) and breeding success. The female fertile phase was assessed following Henderson et al. (2000), assuming female fertility from 5 days before egg laying until the day on which the second-to-last egg was laid. The last fertile day could also have been defined as the day before the laying date of the clutches’ last egg (Supplementary Figure S1). However, since jackdaws usually lay one egg per day (Dwenger 1989; Cramp and Perrins 1994) and it is possible that egg laying occurred after our nest check, we considered Henderson’s definition as more conservative.

### Capture and handling of adult birds

Adult birds were trapped inside nest boxes during the breeding season for individual marking, microphone backpack application, and for collecting biometric data and blood samples. All birds were immediately released after the respective procedures (ca. 20 min including backpack application).

Capture dates were scheduled for each nest box, taking into account the current breeding stage and health state of the respective adults (residents) and their offspring. When possible, capture was carried out when only the target individual was present. Adults were captured either before chick hatching or when chicks were at least 12 days posthatching. If eggs were present in the target nest box, they were temporarily placed in an incubator (for 6 to 32 h) and replaced by false ones to avoid any potential damage. The real eggs were returned to the nest once normal nesting behavior was resumed (assessed via video recordings). Upon reentering the nest box, the females immediately accepted and incubated any false or real eggs.

We captured the adult birds in the nest boxes by pulling a string attached to a trap door. Upon capture, the bird was immediately taken out of the nest box and carried to a nearby quiet working area inside a cotton bag. To reduce stress during handling, a small bag was fitted over the bird’s head. We weighed each bird using a spring balance and measured tarsus length using digital calipers (same experimenter, mean of three measurements). For individual recognition, birds were marked via a unique combination of one aluminum (numbered) and three plastic color rings (Interrex, Łódź, Poland).

### Video surveillance and behavioral analysis

A video surveillance system was installed to ensure the capture of specific target jackdaws under controlled conditions, supervise nesting activities after handling, and to validate classifications of on-bird acoustic recordings in the nest box (see below and see Stowell et al. 2017). These data also provided general nest-box observations (2013–2015, 2017; *n* = 21 nests). However, since the behavior of adult birds was likely affected by handling and backpack attachment (Gill et al. 2016), data for quantifications of behavior in the nest box were collected for 8 of the 16 nest boxes during the otherwise undisturbed prelaying and incubation phase of the main study year (2017).

#### Video surveillance system

The video surveillance system consisted of small, infrared-supported nest-box video cameras with in-built microphones (420 TVL, Handykam, Redruth, UK) connected to a computer with motion-triggered multichannel video surveillance software (GV-1480, GeoVision, Taipei, Taiwan). Motion-triggered recordings were made throughout the early breeding phase from just before sunrise until after sunset. For technical reasons, there were no recordings during the night. For habituation, cameras were installed at least 4 days before the first recordings. Technical issues (e.g., failed automatic PC booting after electricity failure) resulted in some missing recording sessions (2014 and 2015: 3, 0, 1, and 0 per audio-tagged male, see below).

#### Video analysis

Nest-box video analysis was used to record the time individuals spent inside the nest boxes and to detect and quantify instances of sexual and agonistic behavior. All recorded behaviors included date and time (accuracy of 0.5 min) and, if possible, jackdaw ID. The time spent in the nest was calculated for each individual (time of entry minus time of exit) and was summed for each day.

For 2017, motion-triggered video recordings were screened for eight nest boxes (April 4–25). For the 2014–2015 data set (including birds with audio tags), the video footage was prescreened by an experienced technician for sexual behavior and the time at which the birds entered and exited the nest boxes (see Sound analysis). All other video analyses, including identification of individuals, were performed by the first author. In 2017, 18 different jackdaws were individually identified in the video-observed nest boxes. Further birds were identified as unbanded or carrying only one aluminum ring (left or right).

#### Behavior in the nest boxes

##### Nest defense and nest-box intrusions

Residents were defined as the individuals that tended to a clutch in the respective nest box. Because jackdaws may have helpers at the nest (Cramp and Perrins 1994), we paid specific attention to whether the presence of nonresidents was tolerated by the residents. This was never the case, that is, all nonresidents were attacked or driven out of the nest box when the residents were present. Therefore, all nonresident individuals recorded in the nest box in the presence or absence of the resident(s) were defined as intruders.

Nest defense involved attacks, that is, an individual inside the nest-box charging from the current location (usually the nest cup for females) toward the nest-box entrance in a threatening posture (head feathers erect and tail feathers spread (Cramp and Perrins 1994)), or physical fights, sometimes jabbing their beak at potential intruders directly. Nest defense was defined as “joint nest defense” if two individuals (usually, members of a pair) engaged in nest defense simultaneously. Due to the camera’s position inside the nest box, it was not possible to identify individuals that attempted but did not succeed at entering the nest box.

##### Sexual behavior

Based on initial observations and descriptions in the literature (Dwenger 1989), we identified jackdaw copulation behavior, including the female copulation-solicitation posture. We could not define cloacal contact from the videos but distinguished between copulation “attempts” (aborted, wing flapping <2 s) and full “copulations” (not seemingly aborted, wing flapping >2 s), as well as between forced and nonforced sexual behavior (see below). Factors contributing to failed copulations (“attempts”) were external disruptions or a range of avoidance female behaviors (e.g., the female leaving the nest box, not assuming the copulation posture, shaking off the male, stepping away from the nest cup) to physical fights (jabbing the beak at male’s eyes, head, or other body parts, turning around and fighting with claws, physically driving male out of the nest-box).

**Figure 1:**
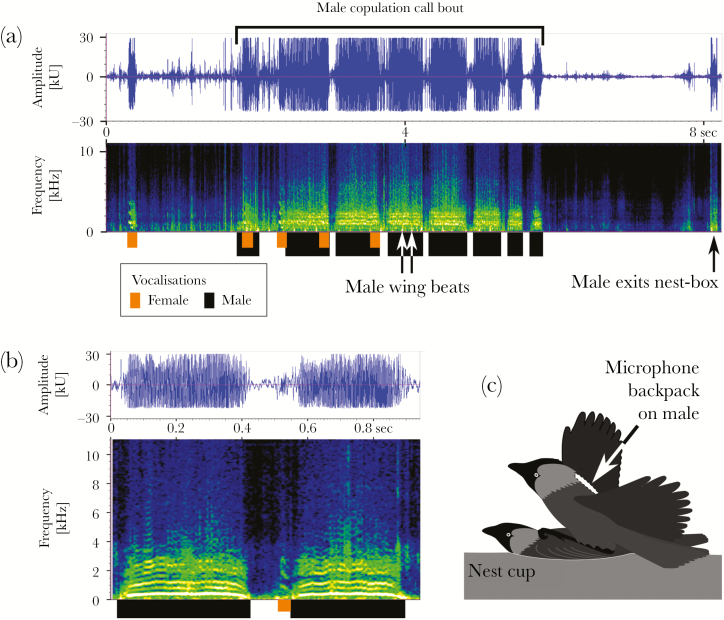
On-board sound recordings during copulation. Waveforms (top; *y* axis: amplitude in kilounits [kU]; *x* axis: time in seconds [sec]) and corresponding spectrograms (bottom; *y* axis: frequency in kilohertz [kHz]; *x* axis: time in seconds [sec]) of (a) an entire bout of copulation calls and (b) three calls: two long copulation calls given by the focal (audio tagged) and one short call given by the nonfocal (nontagged) bird. Due to the position of the backpack on the male (c), the microphone is at a maximum distance from the nonfocal bird’s vocal tract, leading to dampened recordings of female vocalizations (a, b). Thick black and orange bars indicate vocalizations emitted by the male and female, respectively. Male copulation calls were long, harsh (fast amplitude modulation, high energy over large frequency range) and loud, whereas females often produced short calls (a, b). Note additional sounds in the on-board recordings, such as wing beats during copulation and a scraping sound as the male exits the nest box (a; also see Audio 1 and Methods).

The vast majority of sexual behavior took place in the nest cup, with <1% occurring in the nest-box entrance area. For copulation, a male stepped onto the female’s back, stemming its tail feathers on the nest-box floor, pushing its abdomen forward, and flapping its wings (Figure 1c). The female copulation-solicitation posture involved bending the tail upwards and slightly to the side, often accompanied by horizontal tail quivering. Following IP sexual behavior, the male usually rushed to the nest-box entrance and exited or looked out of the entrance hole.

Some copulations were preceded by male and female courtship, that is, horizontal tail quivering and bill-downward postures (Dwenger 1989; Cramp and Perrins 1994), a male’s presentation of a food item or nest material, or were accompanied by soft allopreening of the female head. On the other extreme, sexual encounters could also occur despite female defense behavior. In such cases (forced sexual behavior), an individual entered the nest box rapidly, either by pushing past the female or when the female was turned toward the clutch, facing away from the entrance. These instances were never associated with horizontal tail fanning, bill-down postures, or allopreening. Instead, the intruder immediately mounted the female and began copulation movements without the female adopting the copulation-solicitation posture. Such instances involved the male forcefully holding the female down with the beak, and female defense behaviors, up to severe fights.

### Statistical analyses

The small number of nest boxes with systematic video surveillance (*n*_2017_ = 8) precluded the use of complex statistical models to exhaustively analyze all the collected data. Therefore, we provide simple correlation coefficients, descriptive statistics, or raw values (see Supplementary Figures S1 and S2) and only ran full models if the nature of the data allowed this.

For the statistical models, the observed behaviors (see below) were analyzed in R (R Development Core Team 2018) using a Bayesian statistical approach with uninformed priors and relying on effect sizes instead of *P* values to draw inference. In this framework, meaningful statistical differences are defined to exist between groups if the calculated posterior mean (termed “estimate”) of one group does not overlap with the 95% credible intervals (CrI) of another group. We used mixed-effect models using R package “lme4” (Bates et al. 2015) to account for repeated measures data, including pair identity as a random factor (termed “nest box”). Model assumptions were evaluated by plotting residuals (Korner-Nievergelt et al. 2015). *R*^2^ values, ranging from 0 to 1, were calculated following Nakagawa and Schielzeth, with *R*^2^_marginal_ explaining the variation of the fixed effects and *R*^2^_conditional_ explaining the variation of the fixed and of the random effects (Nakagawa and Schielzeth 2013).

First, we aimed to find out whether male mate guarding was at play. As explained in the introduction, this supposedly costly behavior should be exhibited by males mainly during the female fertile phase and should be reduced during nonfertile periods (Møller and Birkhead 1991; Hunter et al. 1992; Villarroel et al. 1998; Kokko 2005). Further, because, in jackdaws, the females incubate and are courtship fed by males (Dwenger 1989; Cramp and Perrins 1994), nest-box attendance is likely to change differentially for males and females at different breeding stages. Thus, we investigated the time spent by resident females and males inside the nest box, alone or together, across female fertility status (prefertile, fertile, or postfertile). For this, we calculated the time that each resident (*n*_2017_ = 8 females and 8 males) spent inside its respective nest box (*n*_2017_ = 8) per day (*n*_2017_ = 22 days), alone or with its respective partner (“nest-box occupancy”). A mixed-effects model (Gaussian distribution; see Table 1 for model details) was fitted, with the residents’ per-day time spent inside the nest box (log-transformed to improve residual distribution) as explanatory variable, with nest-box occupancy (F: female alone, M: male alone, F + M: female and male together; *n*_2017_ = 175 observations) and resident female fertility status (*n*_prefertile_ = 17, *n*_fertile_ = 73, and *n*_postfertile_ = 85 observations; see Supplementary Figures S1 and S2 for raw data and sample sizes) as two fixed factors and with nest box (*n*_2017_ = 8) as random factor. Because we expected sex-specific differences in the time residents spent in their nest box across female fertility stages, our full model included the interaction term between the two fixed factors, whereas a secondary model included no interaction term (see Table 1). The basic model included no predictors (intercept as fixed effect) but included the random effects (nest-box ID) for intermodel comparability (Table 1).

**Table 1:** Time spent by both partners in the nest-box over changing female fertility status (model 1)

**Model selection**		**Formula**			**AIC**
	Full model (with interaction)	lmer(log(dur + 1) ~ Occupancy × Female + (1|Nest.box))			1448.50
	Secondary model (no interaction)	lmer(log(dur + 1) ~ Occupancy + Female + (1|Nest.box))			1890.50
	Basic model (no predictors)	lmer(log(dur + 1) ~ 1 + (1|Nest.box))			2110.20
**Fixed effects (full model)**	**Female fertility**	**Time spent in nest box by**	**Estimate**	**Lower CrI**	**Upper CrI**
	Prefertile	Female alone	2.35	1.91	2.81
	Fertile		3.48	3.27	3.70
	Postfertile		6.34	6.14	6.53
	Prefertile	Female and male together	5.23	4.77	5.68
	Fertile		5.68	5.45	5.91
	Postfertile		5.21	5.01	5.41
	Prefertile	Male alone	3.56	3.10	4.00
	Fertile		4.12	3.90	4.35
	Postfertile		1.81	1.62	2.02
***R*-squared (full model)**	**Marginal (variation of fixed effects)**	**Conditional (variation of fixed and random effects)**			
	0.72	0.72			

*n* = 525 observations from eight nest boxes (eight females, eight males); female: female fertility status; whos_in: residents in nest box (female alone/female and male together/male alone); CrI: 95% credible interval.

Like mate guarding, high IP copulation frequencies have been associated with costs and should be increased only during the female fertile phase in accordance with the paternity assurance hypothesis (Hunter et al. 1992; Villarroel et al. 1998; Harts et al. 2016). In addition, unless they accrue specific benefits, females often reject their partners’ copulation attempts when they are no longer fertile (Wagner 2010). Thus, we asked whether IP sexual behavior changed with female fertility status by assessing fluctuations in the amount of full copulations versus copulation attempts. For this, we calculated the per-pair (*n*_2017_ = 8; based on data from 22 days) number of full copulations and copulation attempts for each of the three female fertility stages (observations from *n*_prefertile_= 5, *n*_fertile_ = 8, *n*_postfertile_ = 8 nest boxes, respectively; see Supplementary Figures S1 and S2). Then, we fitted a binomial mixed-effect model, with the likelihood of full IP copulations versus IP copulation attempts (counting full copulations as “success” and copulation attempts as “nonsuccess”; see Table 2 for model details) as explanatory variable with respect to female fertility status (see above) as fixed and nest box (*n*_2017_ = 8) as random factor. This full model was compared to the basic model, containing only the intercept as fixed effect and the random factor (for model comparability; see Table 2).

**Table 2:** IP copulations and copulation attempts over changing female fertility status (model 2)

**Model selection**		**Formula**		**AIC**
	Full model	glmer(cbind(nr_cops, nr_atts) ~ Female + (1|Nest.box), family = binomial		138.51
	Basic model (no predictors)	glmer(cbind(nr_cops, nr_atts) ~ 1 + (1|Nest.box), family = binomial)		222.56
**Fixed effects (full model)**	**Female fertility**	**Estimate**	**Lower CrI**	**Upper CrI**
	Prefertile	0.68	0.39	0.88
	Fertile	0.51	0.28	0.74
	Postfertile	0.19	0.08	0.39
***R*** **-squared (full model)**	**Marginal** **(variation of fixed effects)**	**Conditional** **(variation of fixed and random effects)**		
	0.23	0.73		

*n* = 21 observations from eight nest boxes at three different female fertility stages; female: female fertility status; nr_cops: number of copulations; nr_atts: number of copulation attempts; CrI: 95% credible interval.

Next, we wanted to know whether the time spent by females in the nest box alone would predict the occurrences of EP sexual behavior there. Because different amounts of data points were available for the three different female fertility stages and eight nest boxes and the maximum per-day frequency of EP occurrences was three (Supplementary Figures S1 and S2), running a statistical model was not an option. Therefore, we averaged the time each female spent inside her nest box during each fertility stage and calculated the Pearson’s correlation coefficient (*r*), *P* value, and the degrees of freedom (df) for fertile and postfertile females (no EP occurrences in prefertile females).

Lastly, we aimed to find out whether a correlation existed between the occurrences of “incoming” EP sexual behavior (i.e., from the female perspective) and chick-rearing success of a given pair (*n*_2017_ = 8). Since only one data point was available for each nest box, we could not run a complex statistical model with multiple explanatory variables but decided to plot the raw data together with a Pearson’s correlation coefficient (*r*). First, we checked whether the number of EP sexual occurrences was correlated with clutch size because this might bias any correlation with fledging success. Because the Pearson’s correlation coefficient [formula: cor(nr_EPoccurrences, max_eggs)] indicated no correlation (*r* = 0.021, *P* = 0.9606), we then calculated the Pearson’s correlation coefficient, *P* value, and the degrees of freedom of the proportion of chicks that fledged in each of the eight nest boxes [prop_fledged_ = number or fledged/(number of fledged + number of died chicks)] and the number of EP sexual occurrences [cor(nr_EPoccurrences, prop_fledged_)].

### Microphone backpacks, sound recordings, and analysis

As part of a larger project on jackdaw vocal and nonvocal behavior, adult female and male jackdaws were fitted with telemetric equipment, including microphone backpacks (see below) during the breeding seasons of 2014 and 2015. The on-bird audio data set presented here contains all instances of copulation behavior (*n*_2014–2015_=18) detected in four out of five male jackdaws that had been audio-tagged before and during the female egg laying and early incubation phase (no females were tagged from egg laying until chicks were at least 12 days posthatching to avoid the risk of disturbed breeding behavior). The audio logger of the fifth male was damaged and did not contain vocalization or copulation recordings.

#### Microphone backpacks: attachment and settings

Microphone backpacks (6.3–9.5 g, mean: 8.5 g) were attached close to the center of gravity (Vandenabeele et al. 2012; Vandenabeele et al. 2014) on the back of a bird by applying a small amount of flexible glue (Pattex Gel, Henkel, Duesseldorf, Germany) to an area of trimmed dorsal feathers (<5 mm length) and to the backpack (previously sewn to a piece of cloth). These two surfaces were held together firmly for about 20 s. This common attachment method carried the advantage of reducing any risk of injury related to harness methods and avoided the need for recapture because the birds removed the backpacks on their own accord within about a week.

By selecting smaller battery sizes for lighter birds, less than 5% (mean: 3.5% ± 0.5 standard deviation [SD]) was added to each bird’s body mass (205–280 g). Each backpack contained an audio logger (customized digital voice recorders: Edic Mini Tiny A31, TS-Market Ltd., Moscow, Russia) for individual-level sound recordings (Stowell et al. 2017) and a small radio transmitter (BD-2 Holohil, Ontario, Canada) for locating the animals and retrieving the devices (via 5-fold Yagi antenna: F150-151-5FB, Wildlife Materials Inc., Murphysboro, IL; handheld receiver: AOR 8200, AOR, Orange, CA). The audio loggers were customized with a lighter rechargeable battery (ICP581323PA to ICP402035, Renata, Itingen, Switzerland) and shrinking tube casing. They were connected to a PC for charging, setup, and data download (supplemented software: RecManager, version 2.11.19, Telesystems, Moscow, Russia).

Each audio logger was programmed to start 1 day postcapture to avoid potentially decreased vocalization rates after device attachment (Gill et al. 2016). The recording duration was divided across several (5, 5, 5, and 6 per male) consecutive mornings to allow data collection during multiple high-activity periods. Per bird, we recorded 42, 21, 21, and 31 h of continuous onboard sound (22 050 Hz, uncompressed .wav format), with up to 7.5, 5, 5, and 6 h per day, respectively. The technically possible maximum recording duration of 42 h was reduced due to removal by the bird, technical failures, or weight restrictions on batteries for lighter birds.

#### Sound analysis

Sound recordings were acoustically and visually inspected in Audacity (Version 2.0.5) using waveforms and spectrograms (FFT window size 512, Hanning, 0–10 000 Hz, gain 20–35 dB, range 45 dB). Spectrograms presented in the manuscript were created using Raven (Lite 1.0, Cornell Lab of Ornithology). We followed previously described acoustic classification paradigms (Gill et al. 2016; Stowell et al. 2017), for example, to distinguish between the vocalizations of focal (wearing the backpack) and nonfocal (no backpack) individuals to classify call types, including copulation calls to detect copulation behavior of focal individuals and whether focal individuals were inside or outside of a nest box (see below).

##### Focal versus nonfocal vocalizations

Call-type classification was based on previous descriptions (Cramp and Perrins 1994; Dwenger 1989) and observations in the field. To identify the vocalizations of the focal and nonfocal bird, we made use of the microphone’s position on the back of the focal bird and of characteristic sound properties of on-board sound recordings (Figure 1; also see Gill et al. (2016) and Stowell et al. (2017) for details). First, distant background vocalizations were easily separated from foreground vocalizations by pronounced amplitude differences. Second, the vocalizations of focal birds carried more power in low-frequency bands compared to those of nonfocal animals (Ter Maat et al. 2014; Gill et al. 2016). Third, the vocalizations of a focal bird were coupled with body movements that were picked up by the on-bird microphone (Stowell et al. 2017), giving the calls of focal birds a pronounced acoustic onset compared to those of nonfocal individuals. Fourth, focal bird calls, unlike nonfocal calls, were always recorded at the same distance from the bird carrying the microphone. This means that during a continuous sequence of calling, for example, when males and females entered the nest box at different times, only the focal bird remained fixed in the acoustic foreground. Lastly, because jackdaw males mount females for copulation, the microphone on the male’s back was at the largest possible distance from the vocalizing female in this specific context and the female’s vocalizations were attenuated by the bodies of the mating birds (Figure 1).

##### Acoustic copulation detection

Another feature of on-bird microphones is their potential to record more than vocalization data by moving through the same acoustic environment as the animals that carry them (Stowell et al. 2017). Thus, the sound recordings gained from such methods provide additional contextual information that can be extracted by analyzing the characteristic acoustic representations of movement patterns (e.g., flying, scratching) and ambient background sounds (e.g., other vocalizing animals, church bells; Stowell et al. 2017; Greif and Yovel 2019). Here, we exploited this feature to acoustically detect copulations in the on-animal sound recordings. We deliberately chose human-based annotation because experienced human listeners are more efficient at classifying such complex soundscapes than computer algorithms unless a large body of ground-truth data is provided (Stowell et al. 2017). For the annotation process, a human annotator (the first author) was trained in an initial phase on direct observations, and on simultaneous video and sound recordings (also see Stowell et al. 2017) to recognize the acoustic representation of copulations inside a nest box. This was characterized as the following “acoustic scene”: 1) a bird entered a nest box (see below); 2) after a variable amount of time, there were sounds of wing beats and of copulation calls, mostly accompanied by short calls close by; and 3) after a variable amount of time, the bird exited the nest box. The acoustic features of a focal bird being inside as opposed to outside a nest box were 1) a combination of lower overall levels of ambient sounds, slight reverberation of vocalizations, and sounds of the beak, claws, or body touching the wooden nest-box walls and 2) were found in between characteristic loud scraping sounds caused by the microphone brushing against the nest-box entrance hole during entrance and exit (Audio 1).

Following this paradigm, the same experienced annotator screened the spectrograms of all available on-bird sound recordings for sexual behavior, noted down their date and time of occurrence, and extracted these short sound sequences. As mentioned above, for the 2014–2015 data set, an experienced technician screened all available nest-box video recordings for instances of sexual behavior and noted down the respective dates and times. Afterward, the annotator went through all these preselected videos to confirm sexual behavior and to identify the involved individuals. Lastly, the annotator combined the information from the audio and video recordings, which means that the acoustic detection of sexual behavior was performed blindly with respect to IP versus EP behavior.

### Genetics: DNA sampling, extraction, and analysis

#### DNA collection

Forty-four adult birds were DNA sampled in the colony since 2013 (10 buccal swabs, 34 blood samples). For blood sampling, we punctuated the brachial vein using sterile syringe tips and collected up to one capillary of blood per bird. Each sample was immediately transferred to a labeled Eppendorf tube filled with Queens lysis buffer (Seutin et al. 1991) and stored at 4 °C until further processing.

In 2017, we aimed to collect DNA from all chicks hatched in the 16 closely monitored nest boxes (including the eight video-observed ones). To ensure DNA collection of all young despite early chick mortality (Dwenger 1989; Cramp and Perrins 1994) and potential subsequent removal by the parents, we collected DNA via buccal swabs from chicks within 1 day of hatching (Handel et al. 2006; *n*_2017_ = 68; 16 nests). Buccal epithelial cells were obtained by carefully inserting a cosmetic-use cotton tip into the beak of the bird and carefully moving it along the soft tissue (Handel et al. 2006). Each cotton tip was placed into a labeled Eppendorf tube and stored at 4 °C until further processing. One chick died before buccal swabbing and was collected for DNA analysis. The dead chick and unhatched eggs were individually placed into labeled small plastic bags and stored at −20 °C until further processing. We shone a flashlight through the collected unhatched eggs but found no embryo (by naked eye). Because expected amounts of paternal DNA were, thus, extremely low, we did not attempt to extract genetic material from unhatched eggs.

#### DNA extraction, genotyping, and genetic analysis

Total genomic DNA was extracted using extraction kits for blood (NucleoSpin© Blood, MACHEREY-NAGEL, Dueren, Germany) and for tissue (1 mm^2^ tissue sample of clipped toe from frozen chick) and swab samples (innuPREP DNA Mini Kit, Analytik Jena, Jena, Germany).

DNA from buccal swabs successfully amplified for at least 10 of 11 microsatellite loci for 65 of 68 chicks (95.6%, *n*_2017_ = 16 nests). For adults, DNA from all 34 blood samples, but only from 1 out of 10 buccal swabs, amplified successfully (resulting in complete genotypes for 2017 for 5 pairs of social mother and father, 6 social fathers, 9 potential EP fathers, and 10 potential EP mothers). The multilocus exclusion probability for parentage over the 11 analyzed markers, assuming no typing errors, was 0.9992.

Samples were genotyped for 11 microsatellites previously successfully applied to jackdaws (see Supplementary Table S1; Tarr et al. 1998; Haas and Hansson 2008; Wenzel et al. 2011) using a touchdown PCR composed of: initial denaturation at 95 °C for 15 min; 35 cycles of 95 °C for 30 s, the annealing temperature for 60 s, and 72 °C for 60 s; and a final elongation at 72 °C for 30 min. The annealing temperatures for each PCR consisted of two cycles each at 60, 58, 56, 54, and 52 °C and 25 cycles at 50 °C. One microliter of PCR product was added pure (swab samples) or diluted 1:10 (blood samples) to 9 µL Hi-Di formamide and 0.07 µL GS-500 LIZ size-standard (Applied Biosystems, Thermo Fisher Scientific, Waltham, MA) and resolved in POP4 polymer on an ABI 3130 Genetic Analyser (Applied Biosystems, Thermo Fisher Scientific, Waltham, MA) (see Supplementary Table S1 for details). Genotypes were scored using Genemapper v5.0 (Thermo Fisher Scientific, Waltham, MA). Loci were checked for the presence of null alleles, stuttering, and allelic dropout using Microchecker 2.2.5 (Van Oosterhout et al. 2004). A subset comprising all adult individuals caught in 2017 (*n* = 23) was used to assess Hardy–Weinberg equilibrium and linkage disequilibrium using Genepop on the web 4.0.10 (Rousset 2008). Inbreeding Coefficient (G_IS_) and observed and expected heterozygosity were calculated in Genodive 2.0b25 (Meirmans and Van Tienderen 2004). Informativeness for relatedness and multilocus exclusion probability was calculated in KinInfor (Wang 2006).

To determine the proportion of highly related parent individuals, we used Coancestry (v1.0.1.5; Wang 2011) to calculate the degree of relatedness between candidates. Parentage assignment was performed in Cervus (v3.0.7; Kalinowski et al. 2007) using a simulation of 100 000 offsprings, an error rate of 1%, a 95% confidence level, a relatedness of candidate mothers and fathers as calculated by the Coancestry analysis, and conservatively only considered assignments with no mismatches. Additionally, Colony (v2.0.6.2; Jones and Wang 2010) was used to reconstruct sibship relationships among all sampled offspring (parameter settings: typing error rate 1%, male polygyny, long simulation length, full-likelihood method, no updating of allele frequencies, and no sibship size prior). For both analyses, the estimated proportion of mothers and fathers sampled was set to equal the number of sampled social mothers and fathers relative to the total number of sampled nests in the data set.

## RESULTS

### Behavior

#### Social pairs

Twenty-five individually banded jackdaws were observed breeding in the colony during more than one season (maximum 2013–2017). In nine pairs, both members were individually recognized during multiple years, and they were observed breeding with the same partner. In a further seven pairs, one pair member was individually banded and bred with an unbanded individual during multiple years. Field observations and nest-box video footage provided no evidence for trios or of nonpair helpers at the nest. However, nest-box changes from year to year were frequent (79.2%).

#### Breeding stages and resident time spent in the nest box

**Figure 2:**
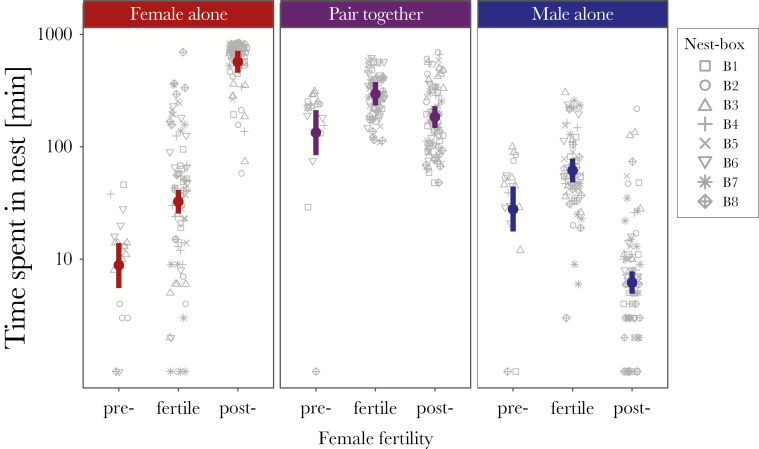
Resident females spent most time alone inside their nest box during their postfertile phase. The time residents spent inside their nests (minutes) per day in relation to female fertility status (prefertile, fertile, and postfertile) and nest-box occupancy (females alone, females and males together, and males alone). Note logarithmic scale for clarity. Left to right panels: females alone, females and males together, and males alone. Gray point symbols: raw data from the eight different nest boxes. Colored thick vertical bars and points indicate the lower and upper credible intervals and the estimates, obtained from the statistical model (see Methods). Statistically meaningful differences exist if there is no overlap between credible intervals and estimates. The time that females spent in their nest alone showed an overall increase in female fertility status. Females and males spent most time together in the nest box when the female was fertile. There was no difference between pair time spent in the nest during prefertile and postfertile phase. Males spent most time in the nest alone during their female’s fertile phase and the least when females were postfertile.

In the main study period (2017, see Supplementary Material for data from further years), female fertility lasted 6–11 days (*n*_2017_ = 16 females; median: 8.8 ± 1.5 SD). For the video-observed residents (*n*_2017_ = 8 females and 8 males from 8 nest boxes; see Supplementary Figure S1 for an overview of time data, female fertile periods, pair-breeding stages, and hatching success), our model showed that the time spent inside the nest box changed with progressing breeding (Figure 2; Table 1). The full model including the interaction term between the two fixed factors (Female and Occupancy) had a lower Akaike information criterion (AIC) than the secondary model where fixed factors were considered separately (AIC_fullmodel_ = 1448.5 vs. AIC_secondarymodel_ = 1890.5; see Table 1). Before female fertility, resident time spent in the nest box was highest for the pair together and lowest for the female alone (Figure 2; Table 1). During the female’s fertile phase, the time spent in the nest box increased for both sexes (alone and together; Figure 2; Table 1). After female fertility, the time spent strongly increased for females and decreased for males (alone and together; Figure 2; Table 1).

#### Nest defense and intrusions

Intruders were nonresident female and male jackdaws (instances involving kestrels and great tits not presented) filmed alone or pairwise (“nonresident pairs”; see Supplementary Material) inside a focal nest box (data from 2013–2015, 2017; *n* = 21 nests). Before, during, and after nest-box intrusions, residents exhibited defensive behaviors alone or jointly, ranging from threatening postures to escalated fights (see Methods). Intruders never entered a nest box if the resident male was inside. If a resident male encountered a nonresident upon returning to its nest box, escalated fights ensued. We found indicators of severe fights in observed and unobserved nests, for example, disarranged nest material, plucked feathers, and drops of blood. We also observed injured adult jackdaws and, in one case, found a female and her 23- and 24-day-old chicks (i.e., after the end of the video-recording period) dead in the nest, all showing signs of severe pecking to vital body parts—indicative of wounds inflicted by one or more conspecifics.

In the eight video-observed nest boxes of 2017, most (1054 out of 1099) intrusions occurred when both residents were absent on average within 39.84 ± 90.45 (mean ± SD) min after the resident male had left the nest and 20.78 ± 60.70 min before his return (excluding nights: 28.04 ± 33.38 and 16.02 ± 24.85 min, respectively). The remaining 45 intrusions occurred when the resident female was in the nest box alone.

#### Sexual behavior

In total, we detected 1414 instances of sexual behavior in the nest-box videos, 1313 of which were recorded in the eight video-observed nest boxes of the main study year 2017 (see Supplementary Material for data from further years). In 2017, 1240 instances were scored as resident IP (94.4%; in all eight nest boxes), 39 as EP (3.0%; in seven nest boxes; i.e., from the female’s perspective), and 34 as nonresident IP (2.6%; in two nest boxes; see Supplementary Material).

**Figure 3:**
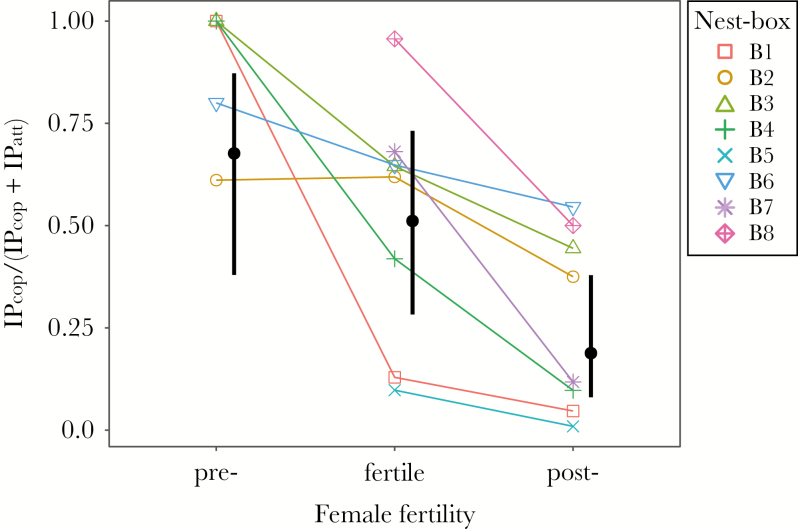
Within pairs, full copulations were increasingly replaced by copulation attempts with progressing breeding. Proportion of full intrapair copulations (IP_cop_) to the sum of full intrapair copulations and intrapair copulation attempts (IP_cop_ + IP_att_) in relation to the resident female’s fertility status (prefertile, fertile, and postfertile). The eight different point symbols and connecting lines represent raw data (summed per pair) for each nestbox. Thick black vertical bars and points: credible intervals and estimates from binomial model (see Methods).

IP sexual behavior began before the resident female’s fertile phase and persisted into the postfertile phase (*n*_2017_ = 8; Supplementary Figures S1–S3). It was observed throughout the day, with a peak in the mornings and evenings (6–10 AM and 5–8 PM; Supplementary Figure S2). Our model showed that, as breeding progressed, IP full copulations were increasingly replaced by IP copulation attempts (*n*_2017_ = 8 nest boxes; Figure 3; Supplementary Figures S2 and S3; Table 2). High levels of variation existed between the resident pairs and their amount of IP sexual behavior (*R*^2^_marginal_ = 0.234; *R*^2^_conditional_ = 0.734; see Table 2 and Supplementary Figures S2 and S3; min = 29, max = 366 IP occurrences).

Unlike for IP sexual behavior, EP occurrences took place during and after but not before the respective resident female’s fertile phase (*n*_2017_ = 8; Supplementary Figures S1–S3). EP sexual occurrences contributed 0–14.7% of all video-observed sexual behavior per resident female (*n*_2017_ = 8) and were scored as copulations in 40–100% of the 39 EP instances (*n*_2017_ = 8 nest boxes; Supplementary Figures S1–S3). We identified at least nine different males engaging in EP sexual behavior, with up to seven different EP males per resident female (*n*_2017_ = 8). Six of them were color banded, and at least three more EP males were observed, but could not be individually recognized (i.e., were unbanded, had only one aluminum ring, or the color bands were not identifiable). EP sexual behavior occurred throughout the day, often during the morning and evening hours, and it is worth noting that, out of the 39 EP sexual occurrences, 10 took place before the resident male’s return to the nest box in the morning and 6 after his last visit in the evening (*n*_2017_ = 8 nest boxes; Supplementary Figure S2).

The Pearson’s correlation coefficient of the time females spent inside their nest boxes alone and the occurrences of EP sexual behavior was 0.5937 for fertile (*n*_2017_ = 3 occurrences in 2 out of 8 females; *P* = 0.1208, df = 6) and 0.3135 for postfertile females (*n*_2017_ = 36 occurrences in 7 out of 8 females; *P* = 0.4495, df = 6). This suggests that females that spent more time alone inside their nest boxes might be more likely to experience EP sexual behavior there during specific breeding stages, but a larger sample size is required to verify this statistically.

In all 73 observed instances of EP sexual behavior (*n*_2017_ = 39 occurrences in 7 out of 8 pairs; *n*_2014 + 2015_ = 34 occurrences in 7 out of 9 pairs, see Supplementary Material for details), a nonresident male entered the focal nest box and performed sexual behavior with the resident female. None of these instances involved female copulation solicitation, and we found no evidence for females actively seeking EP sexual behavior. In the vast majority of cases, the female engaged in nest defense before the nonresident entered the nest box (except for a few instances, *n*_2017_ = 2, in which the resident female was seemingly asleep or with her back to the entrance). No instance of EP sexual behavior was initiated in the presence of the resident male.

#### Acoustic events and copulation calls

From our direct observations and video recordings, we found that the observed IP and EP copulations were always accompanied by the loud, long, and harsh copulation calls. Detailed acoustic analyses were performed on the on-bird sound recordings available for four out of the five different males (data from 2014 and 2015; see Methods for sample sizes). All individually recorded copulation call bouts (Figure 1; *n*_2014–2015_ = 18; with 11, 1, 4, and 2 copulations per male) were produced by the audio-tagged bird, that is, the respective male, and always occurred inside a nest cavity, whereas the nonfocal bird close by (presumably the female copulation partner) often produced short calls (Figure 1; Audio 1). Overall, copulation calls lasted 0.11 to 1.10 (0.51 ± 0.14 SD) s and occurred in bouts of 3.42 to 59.60 (19.45 mean ± 20.35 SD) s (Supplementary Table S2).

For these 18 instances of acoustically detected male copulation behavior, 14 instances of simultaneous video recordings of the focal bird’s nest box were available (in four cases, recordings were missing for technical reasons; 3, 0, 1, and 0 cases per male). In 11 cases, the videos showed (intra)pair copulation behavior in the focal bird’s nest box (6, 0, 3, and 2 copulation videos per male). In the remaining three instances (2, 1, 0, and 0 per male), the video showed the resident female inside the focal bird’s nest box alone, suggesting the focal male engaged in EP sexual behavior outside of its own nest box. It is worth noting that during these instances of presumed EP sexual behavior, the audio-tagged male appeared to have changed its vocal behavior compared to during instances of IP sexual behavior, increasing the mean bout duration and number of calls (see Supplementary Table S2). Unfortunately, these three instances of male behavior did not take place in any of the other nest boxes with video surveillance, and the small sample size does not allow more detailed analysis.

### Breeding success and genetics

#### Breeding success

Overall, the colony’s clutch size was between 2 and 8 eggs per nest (see Supplementary Material). In the main study year 2017, 78 eggs were laid (median: 5 ± 1.15 SD; *n*_2017_=16 nests). 69 of them hatched (88.5%; in all 16 nests), 7 (9% of total colony; in 5 nests) failed to hatch, and 2 disappeared during the egg-laying period (in 1 nest). Ten chicks fledged (14.5%; in eight nests). Chicks that did not fledge died at an average age of 11.2 days posthatch (*n*_2017_ = 59; median: 8 ± 9.6 SD days).

**Figure 4:**
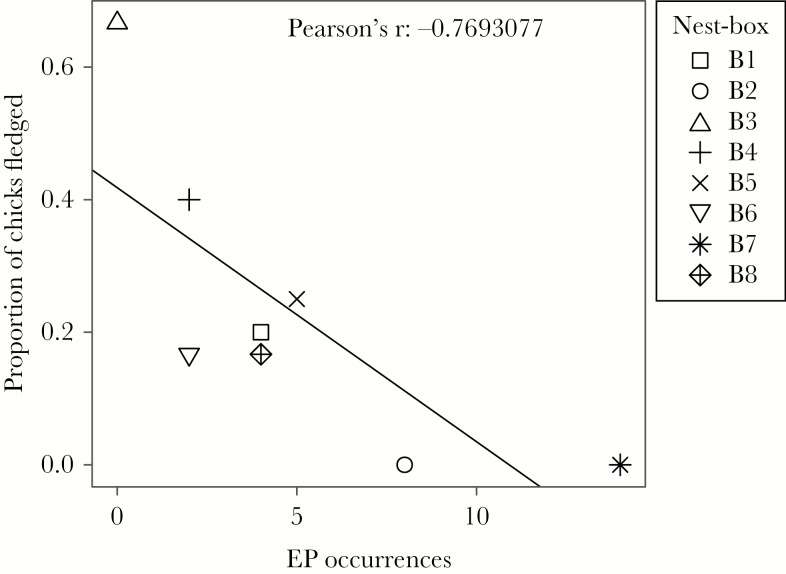
Negative relationship between the occurrences of EP sexual behavior and chick-rearing success. The proportion of fledged chicks [*n*_chicks fledged_/(*n*_chicks fledged_ + *n*_chicks died_)] and the total number of EP sexual occurrences were negatively correlated (Pearson’s correlation coefficient: −0.769, *P* = 0.0256, df = 6; *n* = 8; point symbols: raw data from the eight different nest boxes).

For the eight video-observed nest boxes of 2017, hatching and fledging success was 85.3% and 20.7%, respectively. Eggs failed to hatch in three of the eight nest boxes, one with the highest (B7), one with medium (B5), and one nest box without any occurrences of EP sexual behavior (B3; Supplementary Figure S1). We found no correlation between clutch size and the amount of incoming EP sexual behavior (i.e., involving the resident female and a nonresident male; *n*_2017_ = 8; Pearson’s coefficient = 0.021, *P* = 0.9606, df = 6). However, for the same nest boxes, there was a negative association between the amount of incoming EP sexual behavior and fledging success (the proportion of fledged chicks per nest; *n*_2017_ = 8; Pearson’s coefficient = −0.769, *P* = 0.0256, df = 6; Figure 4). Eggs were considered potentially fertilized by an EP male if, at least, one EP copulation occurred during female fertility, at least, 1 day before the recorded laying date. Such potential EP-fertilized eggs were found in two of the eight video-observed nest boxes, and both of these eggs hatched (nest boxes B2 and B8; Supplementary Figure S1).

#### Parentage and sibship

Parentage assignment in Cervus identified the social parents as the genetic parents for all offspring for which one or both parent genotypes were available, including for all eight video-observed nest boxes (*n*_2017_ = 8 female, 8 male residents). No cases of assignment to either a potential EP father or mother, or to the social father or mother of a different nest, were observed in any of the offspring. Sibship reconstruction in Colony yielded strong support for full-sib relationships between all offspring in 15 out of 16 nests. In the remaining nest, only two out of five chicks were assigned as full sibs, suggesting the possibility of EP paternity. However, since this nest was unmonitored and neither of the social parents was sampled, this assignment remains speculative.

## DISCUSSION

### Mismatching social, sexual, and genetic monogamy

Recent studies of avian monogamy focused mainly on its genetic aspects but largely lacked assessments of actual sexual behavior. First, genetics provide quantifiable insight into sexual selection and conflict (Westneat and Stewart 2003; Kempenaers and Schlicht 2010; Chaine et al. 2015) and, second, keeping track of avian copulation behavior remains challenging (Kempenaers and Schlicht 2010). We used a multilevel approach to quantify and directly compare the social, sexual, and genetic aspects of monogamy in wild jackdaws, a rare example of “strict monogamy” in birds (Henderson et al. 2000). Our data confirmed social monogamy (Roell 1978; Dwenger 1989; Cramp and Perrins 1994) and supported previous evidence for high-level genetic monogamy (Liebers and Peter 1998; Henderson et al. 2000). However, EP sexual activity was found in almost all observed pairs, thus challenging the much-cited “strict monogamy” (Henderson et al. 2000) at the sexual level. In the following, we discuss potential explanations for this mismatch between behavior and genetics.

#### Sampling bias

First, the absence of EP offspring reported in previous studies could be explained by methodological differences. Blood samples of altricial bird chicks are not usually collected immediately after hatching (Liebers and Peter 1998; Fair et al. 2010; Handel et al. 2006; Salomons et al. 2009) but, in jackdaws, young nestling mortality is high, especially for late-hatched chicks (Dwenger 1989; Cramp and Perrins 1994). Further, avian EP copulation rates may differ systematically with egg-laying order (Griffith et al. 2002; Krist et al. 2005; Vedder et al. 2012) and our observations suggested increasing amounts of EP sexual behavior over time. If so, late-hatched chicks would be more likely to be EP young, as shown for other birds (Safari and Goymann 2018), and might have died by the time they were scheduled for blood sampling, in previous studies. Our sample size was on a similarly small scale as the available studies combining jackdaw parentage and behavior (Liebers and Peter 1998; Henderson et al. 2000). However, having marked all eggs and collected chick DNA via buccal swabs (Handel et al. 2006) and tissue of dead chicks, we were able to sample the DNA of all hatched chicks (*n*_2017_ = 69) in the available 16 clutches and to quantify the number of unhatched or missing eggs and chicks. We can, thus, rule out systematic sampling bias as an explanation for the missing EP offspring in our study.

#### Fertilization, hatching, and fledging rates

In birds, a variety of postcopulatory selection mechanisms may alter the genetic outcome of sexual behavior by affecting fertilization, hatching, or fledging rates (Griffith et al. 2002; Kempenaers and Schlicht 2010; Birkhead 2010). In our study, we found two potentially EP-sired eggs (in nest boxes B2 and B8; see Supplementary Figure S1), but both of them hatched and were genetically assigned to the social father with no mismatches. As mentioned above, it is possible that, in jackdaws, EP-sired eggs are more likely to occur later in the laying sequence and that EP chicks may, thus, show higher mortality than IP chicks. However, the data available to date does not allow making any predictions about systematic differences between EP and IP fertilization or hatching rates, chick-rearing conditions, or fledging success.

#### Mate guarding and frequent IP copulations

Birds have evolved a number of behavioral strategies to prevent EP fertilizations. By staying close to their mate, the partner decreases the likelihood of EP copulation occurrences (mate guarding), thereby monopolizing genetic parentage and reducing the risk of sexually transmitted diseases (Kempenaers and Schlicht 2010). Despite potentially missed EP opportunities, the guarded partner may also profit from this behavior, for example, through protection from harassment (Kempenaers et al. 1995). Another behavioral parentage assurance strategy is frequent IP copulation, which may dilute potential EP competitor’s sperm and make EP fertilization less likely (Møller and Birkhead 1991; Bertran et al. 2016; Harts et al. 2016). One or both partners may further profit from frequent IP copulations through direct benefits, for example, food items or harassment protection, intensified pair bonding, partner quality assessment, or territorial signaling (Venier and Robertson 1991; Villarroel et al. 1998; González-Solís et al. 2001; Negro and Grande 2001). Yet, both mate guarding and frequent IP copulations are considered costly as they may increase energy demands or predation risk and reduce the time allocated to foraging or seeking EP opportunities (Møller and Birkhead 1991; Kokko 2005; Harts et al. 2016). Thus, if these behaviors were part of a parentage assurance strategy, they should be expressed mainly during the female fertile period (Villarroel et al. 1998).

In our study, both behaviors were likely at play and could—at least in part—explain the absence of EP offspring. First, EP sexual behavior never occurred when the resident male was present, and resident males spent most time in their nest boxes during their female partner’s fertile phase. Hence, EP sexual behavior rarely occurred during female fertility (*n*_2017_= 3 out of 39 occurrences in 2 out of 8 nest boxes). Next, frequent IP sexual behavior outnumbered by far EP sexual occurrences. As predicted by the paternity assurance hypothesis (Villarroel et al. 1998), both behaviors peaked around female fertility and, once females were no longer fertile, males spent less time inside the nest box, IP sexual behavior decreased, and EP sexual behavior increased. Whether the latter was driven by the resident male’s absence or by the fact that other males simultaneously reduced their time allocated to mate guarding in favor of seeking EP sexual opportunities could not be disentangled and are not mutually exclusive.

#### Female behavior

Although females of some bird species may profit from continued copulations even after the fertile phase, females of other species may reject copulations with their partner early on (Wagner 2010). This has been attributed to different factors, for example, sperm competition (rejecting IP in favor of EP copulations), specific forms of mate guarding, and shorter or less female motivation to copulate (Wagner 2010). In many bird species, females may enhance their fitness by seeking EP copulations (Double and Cockburn 2000; Kempenaers and Schlicht 2010), despite the benefits of mate choice and long-term pair bonds (Sanchez-Macouzet et al. 2014; Ihle et al. 2015). In our study, females rejected both IP and EP copulations after the fertile period, suggesting that they do not profit from extended copulation behavior neither with their own partner nor with an EP male. In fact, we found no evidence for females seeking EP copulations at all (in theory, they could have taken place outside of the observed nest boxes, but there was no evidence that jackdaws copulate outside of the nest cavity; also see Dwenger (1989), Cramp and Perrins (1994), and Liebers and Peter (1998)). Instead, while IP sexual behavior was never associated with escalated aggression, even though females increasingly rejected their partners’ copulation attempts over time, EP sexual behavior appeared to be forced because it never involved the female copulation-solicitation posture and female nest-defense behavior and physical resistance to the EP male were prevalent. In other species, within-pair sexual behavior is often less aggressive and more likely to involve courtship than in EP contexts (Tarof and Ratcliffe 2000). Further, in many bird species, males do not possess a real intromittent organ and females can control cloacal contact, sperm transfer, storage, or ejection (Lifjeld and Robertson 1992; Birkhead and Møller 1993; Briskie and Montgomerie 1993; Pizzari and Birkhead 2000). Concurrently, forced EP copulations have been shown to result in systematically low EP fertilization rates in some socially monogamous species (Emlen and Wrege 1986; Dunn et al. 1999) and could explain the lack of EP offspring in our study. However, since only few instances of EP sexual behavior occurred during the female fertile phase, this notion requires further study. Future studies should also investigate potential explanations for why females seemed to avoid EP copulations, such as the absence of genetic benefits, for example, via female-driven mate choice or the presence of specific costs.

### Costs and benefits of EP behavior

#### Costs of EP sexual behavior

Depending on the species, avian EP sexual behavior has been described not only as a costly byproduct of colony living but also as a potential driver of coloniality. In the latter case, even in socially monogamous species, close-by EP males could attract females in a lek-like system where EP males compete and provide high-quality sperm but not any parental care (Wagner 1998). In this system, females either actively seek copulations or show high numbers of EP fertilizations through high-quality males (Wagner 1998). Yet as explained above, females did not seem to cooperate during EP copulations and did not profit from them in terms of high-quality EP offspring. As in previous work (Liebers and Peter 1998), we too found some evidence for potential EP offspring (2017: 1 out of 16 nests), although at a much lower rate than the observed rate of EP sexual behavior would suggest (2017: in 7 out of 8; 2014 + 2015: in 7 out of 9 nest boxes). Thus, EP sexual behavior may prove a beneficial alternative reproductive strategy over time or under specific ecological circumstances, for example, in situations where resident male mate guarding is reduced due to low food availability, resulting in extended foraging trips. However, our observations also suggested that jackdaw EP copulations might be associated with high costs. For instance, EP sexual behavior appeared to be physically dangerous for all individuals involved because it carried a risk of injury and infection, for example, during female physical defense or severe fights with the returning resident male. In fact, the timing of EP sexual behavior suggested that EP males sought to minimize the chance of encountering the resident male, for example, by entering nest boxes before the resident male’s return from, or respectively after its departure to, the communal night roost (up to 35 km from the breeding colony; Cramp and Perrins 1994; here, ca. 12 km linear distance). Since collective behavior such as communal roosting or foraging is beneficial to individuals of group-living species (Bijleveld et al. 2000; Wright et al. 2003; Evans et al. 2016), leaving the group to seek EP sexual opportunities may also come at a cost in terms of increased predation risk and reduced foraging opportunities (Kempenaers and Schlicht 2010). Because all evidence collected on jackdaws so far indicated zero to low levels of EP fertilization rates, and costs might be high, EP copulations are not as easily explained via increased individual fitness benefits, unlike in many other avian species (Double and Cockburn 2000; Kempenaers and Schlicht 2010).

#### Copulation calls as territorial or dominance signal?

In raptors, frequent and conspicuous copulations in open nests may signal territoriality (Negro and Grande 2001; Martínez et al. 2019). Although jackdaw copulations occur visually concealed inside nest cavities, they too are rather conspicuous because of the associated loud copulation calls (Dwenger 1989; Cramp and Perrins 1994). Given the potential costs of EP sexual behavior discussed above, it is intriguing that EP males did not suppress this vocal behavior. In songbirds, vocalizations often signal territoriality (Catchpole and Slater 1995), and there is growing evidence that jackdaws recognize individual conspecifics’ vocalizations (Lorenz 1931; Dwenger 1989; Zandberg et al. 2014; Stowell et al. 2016; Woods et al. 2018). A recent study suggested that simulated male EP copulation calls elicited behavioral responses in the “cheated upon” female partners (Lee et al. 2019). Like in other species, jackdaw copulations could, thus, be used to advertise and detect copulation behavior (Hsu et al. 2006; Montgomerie and Thornhill 2010; Schlicht and Kempenaers 2016) and may provide identity-associated information, for example, to signal nest-site occupation by a mating pair. Jackdaws spend a large proportion of the year disputing over nest sites and may engage in severe fights over this resource (Roell 1978; Cramp and Perrins 1994). Loud male calls during sexual behavior inside an EP nest may lay claim to additional or future nesting sites, that is, to increase the size of a male’s territory acoustically. Lastly, due to the loud copulation calls, a resident male returning to the colony would be likely to detect EP sexual behavior occurring in its nest before entering, which might affect its decision-making process, for example, whether to attack this specific intruder or not. Copulation calls may, thus, also function as a dominance signal as has been shown, for example, in primates (Hauser 1998).

#### Do frequent EP occurrences lower breeding success?

Although based on a small sample size, the negative association between the frequency of female EP sexual encounters and chick fledging success remains intriguing. Clearly, further data are necessary to investigate whether this pattern would be reproducible given a larger sample size and under different socioecological parameters, such as colony breeding density and synchrony or the immediate proximity to neighboring nests (Hoi and Hoi-Leitner 1997; Griffith et al. 2002; Westneat and Stewart 2003; Kempenaers and Schlicht 2010; Brekke et al. 2013). In any case, it raises the question whether jackdaws may directly profit from reduced fledging success of their competing colony mates or whether it may rather reflect a byproduct of pair dominance or quality. If the latter were the case, the negative association could be explained by the fact that those pairs that were attacked more, or were less efficient at defending their nests, were also the ones that were less successful at raising their nestlings. Our data do not provide information on the colony’s dominance hierarchy, but we found that EP sexual behavior was not only carried out by unsuccessful individuals (e.g., without own nest box or with a failed clutch) as data from focal observations had suggested (Liebers and Peter 1998; but see Schuett et al. 2012). Judging from our observations, it is quite possible that eggs were damaged during nest defense or the forceful nest-box intrusions. Also, since EP sexual behavior seemed highly stressful to females, any negative association with chick fledging could be explained by long-term negative effects during incubation or chick rearing. Alternatively, engagement in EP sexual behavior may be detected by the partner, via the unmistakable, loud copulation calls, and “punished” via reduced parental care (Valera 2003). Given that jackdaw females seem to respond to simulated EP copulation calls (Lee et al. 2019) and resident males sometimes interrupted EP sexual occurrences in our study, this is conceivable but seems rather unlikely in a species with lifelong social pair bonds and biparental care (Lorenz 1931; Roell 1978; Dwenger 1989; Cramp and Perrins 1994; Liebers and Peter 1998; Henderson et al. 2000). However, since we only video-recorded behavior in the nest boxes until around chick hatching, future research is required, involving male and female provisioning rates in relation to EP sexual behavior.

## FUNDING

This work was supported by the Max Planck Society.

## Supplementary Material

arz185_suppl_Supplementary-Material

arz185_suppl_Supplementary_Audio-1

## Notes

We thank Magdalena Pelayo van Buuren, Magdalena Mair, and Thibault Boelhy for field assistance, Ingrid Schwabl for initial video coding, Wolfgang Goymann for input and support, Moritz Hertel and Antje Bakker for assistance in the lab, Gerald Kerth for providing lab facilities, and the von Bayern family for access to the jackdaw colony.

Data accessibility: Analyses reported in this article can be reproduced using the data provided by Gill et al. (2019).
