# Supplementary material for: Genetic monogamy despite frequent extrapair copulations in “strictly monogamous” wild jackdaws
Source: Behav Ecol. 2019 Nov 22;31(1):247–60. doi: 10.1093/beheco/arz185 (PMC7191249; doi:10.1093/beheco/arz185)
Supplement: arz185_suppl_Supplementary-Material [file arz185_suppl_supplementary-material.docx]

# SUPPLEMENTARY MATERIALS

### Remaining dataset

In three breeding seasons preceding the main observation period of 2017, between 3 and 8 eggs were laid per clutch (median 5 ± 0.9888265). The overall estimated success of hatching was 78, 75 and 85%, and of fledging was 21, 31 and 13%, respectively.

In the preliminary nest-box video recordings (collected in 2014 and 2015), we detected 101 instances of sexual behaviour in 9 different nest-boxes. 65 were IP (in 8 nest-boxes), 34 were EP (in 7 nest-boxes), and two were unknown. EP sexual behaviour involved up to 5 different EP males per resident female. We identified 2 colour-banded EP males for 2014, and 4 for 2015. In both years, we found at least three more EP individuals that could not be identified further (unbanded, one Alu ring, colour-bands unidentified).

### Non-resident pairs

At the beginning of the breeding period, activities of non-resident pairs included nest-building, intra-pair allopreening and sexual behavior (Nest-boxes B2-B4, Fig. S2), as well as nest defense behavior (data not presented). Note that occurrences of non-resident intra-pair sexual behavior only occurred before the resident females were egg-laying, and occurred mostly in the middle of the day (Fig. S2). Later during breeding, non-resident activities in the focal nest-boxes included removal of nest material, fights and extra-pair sexual behavior (data not presented), but no longer included intra-pair sexual behavior (Fig. S2).

# Supplementary Materials – Figures:


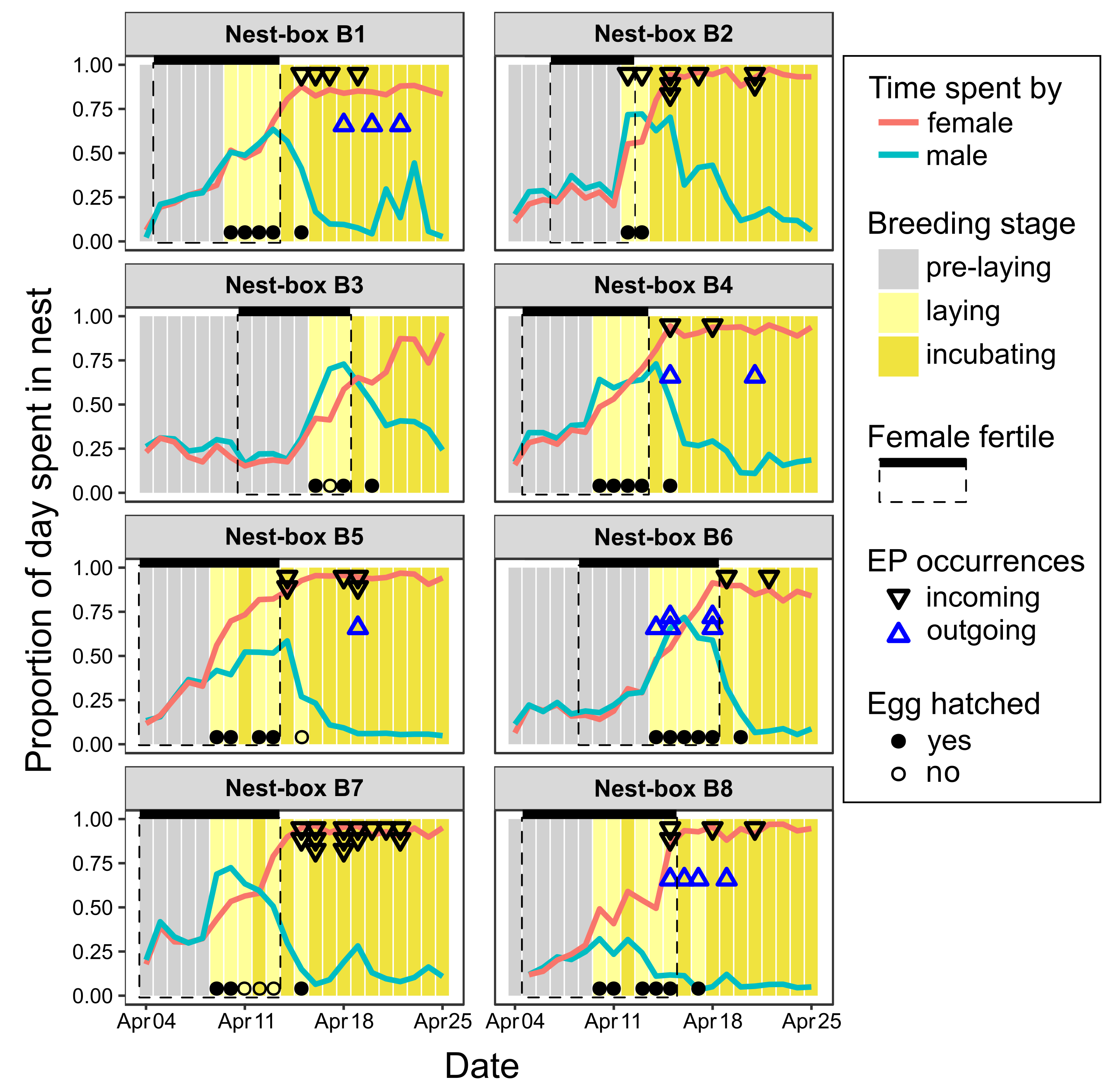


**Figure S1: Nest-box occupancy and extra-pair (EP) sexual behavior over breeding stages.** This figure provides, for each observed nest, a day-by-day overview of each resident’s nest-box attendance (proportion of recorded time per day; red lines: females, turquoise lines: males), the resident pairs’ breeding stages (grey and yellow bars), the female fertile phase (black bars), and the occurrence of EP sexual behavior. Black downward-pointing triangles represent EP sexual behavior involving a non-resident male detected in the focal nest-box (“incoming”). Blue upward-pointing triangles indicate EP sexual activity of a resident male of a focal nest detected in one of the seven other filmed nests (“outgoing”). Laying date and hatching success (asterisk) and hatching failure (point) of each egg is indicated at the bottom of each plot.

**
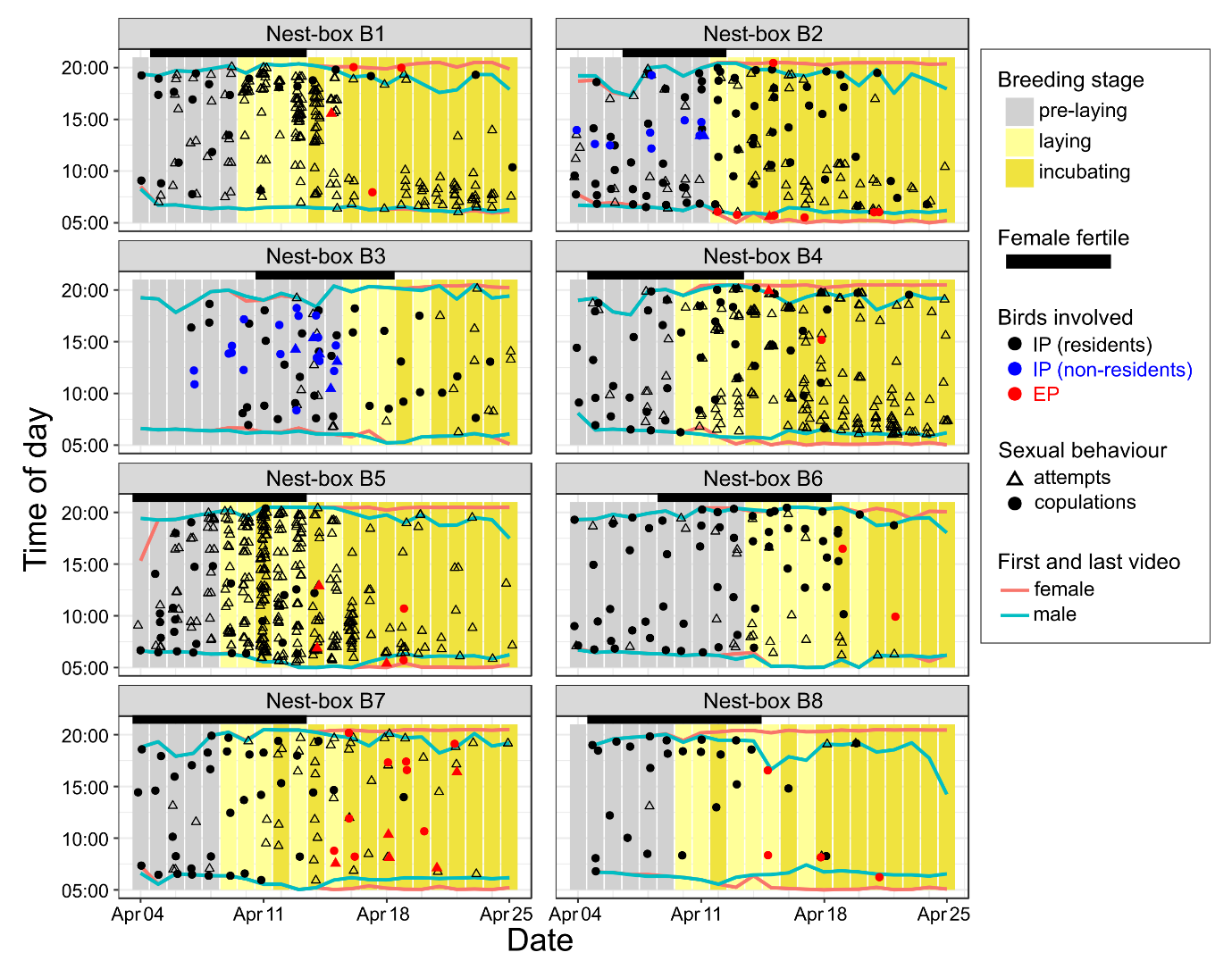
**

**Figure S2: The occurrences of all observed sexual behaviour during the course of the day plotted for each date and nest-box.** Coloured vertical bars indicate breeding stages, and thick black lines female fertile periods (see Fig. 1). Red (female) and turquoise (male) lines indicate the time of first and last observation per day and resident. Coloured points (copulations) and triangles (attempts) indicate sexual behaviour involving the resident female with the resident male (black: IP residents) or with a non-resident male (red: EP non-resident), or involving a non-resident pair (blue: IP non-residents). Resident IP copulations occurred mostly in the morning and evening hours, and were increasingly replaced by copulation attempts as breeding progressed (see Fig. 4). Non-resident IP sexual behaviour occurred mostly during midday, and ceased when resident females began egg-laying. EP sexual behaviour was detected only during the egg-laying and incubation phase of the resident female. 10 instances of EP sexual behaviour occurred before the resident male’s first, and 6 after his last daily visit to the focal nest-box.

**
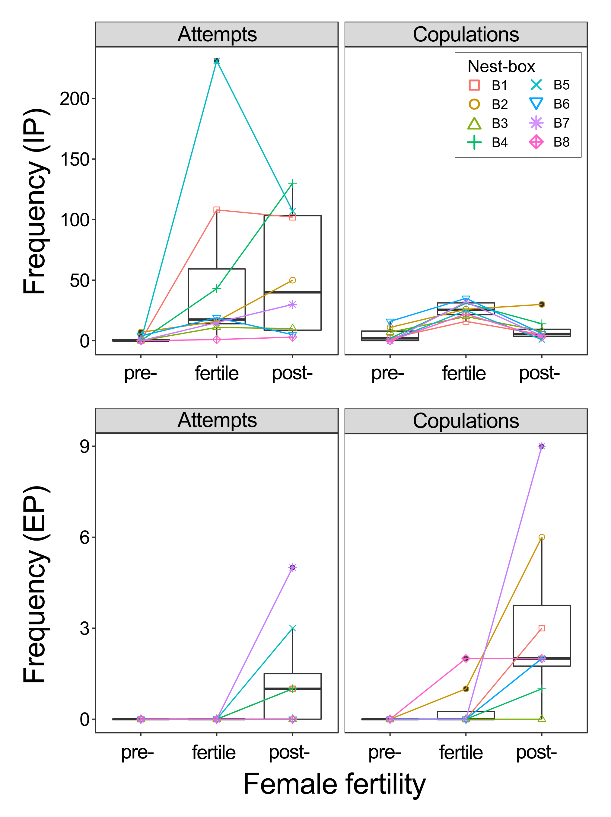
**

**Figure S3: Extra-pair and intra-pair sexual behaviour changes differentially with female fertility.** Raw data (coloured points and connecting lines per nest-box) and box plots (centre line: median; upper and lower margins: 25th and 75th percentiles; whiskers: 1.5x inter-quartile range; filled black shapes: outliers) of occurrences of intra-pair (IP, above) and extra-pair (EP, below) sexual behaviour), split into “attempts” (left) and “copulations” (right), in relation to the resident female’s fertility status (pre-fertile, fertile, post-fertile. Within pair, copulations were increasingly replaced by attempts. Note the difference in scale between IP and EP frequency. EP sexual behaviour occurred in 3 cases during the female fertile, and in 36 cases in the post-fertile period.

# Supplementary Materials – Tables:

| **Name** | | **Primer Sequence** | **Ascension Number / Source** | **Multiplex / T_anneal_ / Concentration / Dye** | | **No. of Alleles** | | **Size Range** | | | **H_O_** | | **H_E_** | **G_IS_** | | **Ir** | **P_HWE_** |  |
| --- | --- | --- | --- | --- | --- | --- | --- | --- | --- | --- | --- | --- | --- | --- | --- | --- | --- | --- |
| **Ck.1B6G** | F: ATGGAGTGGAGGGAGAGATG | | AF026333 | | A / TD_60-50_ | 4 | | 116-132 | | | 0.68 | | 0.675 | -0.007 | | 0.090 | 0.582 |  |
|  | R: AGTACTACCAGTTCACCTGC | | (Tarr et al. 1998) | | 1µM / PET |  | |  | | |  | |  |  | |  |  |  |
| **Ck.4B6D** | F: TTGCATCCCTGATTTATGGC | | AF026340 | | A / TD_60-50_ | 4 | | 114-146 | | | 0.28 | | 0.291 | 0.037 | | 0.038 | 0.483 |  |
|  | R: CTAGGAAGCAATCCAGAGTC | | (Tarr et al. 1998) | | 0.5 µM / VIC |  | |  | | |  | |  |  | |  |  |  |
| **Ck.5A5F** | F: GTGGTTATACCAGAGGTCCT | | AF026338 | | A / TD_60-50_ | 11 | | 152-176 | | | 0.92 | | 0.894 | -0.029 | | 0.166 | 0.494 |  |
|  | R: TTTTGTTCTCTCAAGACACC | | (Tarr et al. 1998) | | 1µM / FAM |  | |  | | |  | |  |  | |  |  |  |
| **Ppi2** | F: CACAGACCATTCGAAGCAGA | | AJ272375 | | B / TD_60-50_ | 5 | | 239-251 | | | 0.56 | | 0.507 | -0.103 | | 0.071 | 0.335 |  |
|  | R: GCTCCGATGGTGAATGAAGT | | (Haas and Hansson 2008) | | 0.5 µM / VIC |  | |  | | |  | |  |  | |  |  |  |
| **Ppy-01** | F: TCCCAACAAAGCAACAAACA | | JF304556 | | B / TD_60-50_ | 3 | | 165-169 | | | 0.40 | | 0.439 | 0.089 | | 0.051 | 0.389 |  |
|  | R: TGGCAAAAACGAAAGACTAGC | | (Wenzel et al. 2011) | | 1µM / NED |  | |  | | |  | |  |  | |  |  |  |
| **Ppy-04** | F: CCTTGCTGTCTGTTCAAATAA | | JF304559 | | B / TD_60-50_ | 4 | | 150-162 | | | 0.24 | | 0.255 | 0.059 | | 0.033 | 0.387 |  |
|  | R: TTGGCATGCATGAAATTTGT | | (Wenzel et al. 2011) | | 0.5 µM / FAM |  | |  | | |  | |  |  | |  |  |  |
| **Ppy-05** | F: CTGTCTCCCAGCAGAGAACC | | JF3045560 | | A / TD_60-50_ | 7 | | 199-227 | | | 0.72 | | 0.733 | 0.018 | | 0.111 | 0.513 |  |
|  | R: TCGCTCCATGCTTTTATTCC | | (Wenzel et al. 2011) | | 0.5 µM / FAM |  | |  | | |  | |  |  | |  |  |  |
| **Ppy-08** | F: AGAGAGATTTTACCATGGGAGAT | | JF304563 | | A / TD_60-50_ | 7 | | 223-247 | | | 0.88 | | 0.831 | -0.059 | | 0.132 | 0.367 |  |
|  | R: AGACTGATTGCCGGACTTTG | | (Wenzel et al. 2011) | | 0.75 µM / NED |  | |  | | |  | |  |  | |  |  |  |
| **Ppy-13** | F: AGCTCACTTCTTGCTCACAGTTT | | JF304568 | | A / TD_60-50_ | 7 | | 180-204 | | | 0.76 | | 0.779 | 0.025 | | 0.115 | 0.492 |  |
|  | R: GCTTCAGGCTGTTCTATCTATC | | (Wenzel et al. 2011) | | 0.5 µM / VIC |  | |  | | |  | |  |  | |  |  |  |
| **Ppy-14** | F: GGCCTTGAAAGAAGTGTGCT | | JF304569 | | B / TD_60-50_ | 15 | | 298-346 | | | 0.92 | | 0.931 | 0.012 | | 0.166 | 0.518 |  |
|  | R: GCCTGATCCTCTTCTTGCTTT | | (Wenzel et al. 2011) | | 1.5 µM / PET |  | |  | | |  | |  |  | |  |  |  |
| **Ppy-15** | F: CTTTCATCAGCAGGCGATCT | | JF304570 | | B / TD_60-50_ | 5 | | 142-160 | | | 0.64 | | 0.605 | -0.058 | | 0.078 | 0.434 |  |
|  | R: GTTGTCCAATGGAAGGCATC | | (Wenzel et al. 2011) | | 0.75 µM/PET |  |  | |  |  | |  | |  |  | | | |

**Table S1:** Characterisation of the microsatellite loci and their amplification conditions. Observed heterozygosity (HO), expected heterozygosity (HE), Nei’s inbreeding coefficient (GIS), and the P-value of the test of Hardy-Weinberg equilibrium (PHWE) were calculated in Genodive 2.0b25. Informativeness for relatedness (Ir) was calculated in KinInfor (see main text).

|  | **Total** | **Within pair** | **Extra pair** | **Bird** |
| --- | --- | --- | --- | --- |
| **Number of bouts** | **18** | **11** | **3** | **All** |
|  | 11 | 6 | 2 | bird1 |
|  | 1 | 0 | 1 | bird2 |
|  | 4 | 3 | 0 | bird3 |
|  | 2 | 2 | 0 | bird4 |
| **Total number of calls** | **578** | **359** | **115** | **All** |
|  | 147 | 48 | 24 | bird1 |
|  | 37 | 0 | 37 | bird2 |
|  | 344 | 264 | 0 | bird3 |
|  | 47 | 47 | 0 | bird4 |
| **Mean nr of calls per bout ± SD** | **31.94 ± 32.46** | **32.64 ± 37.08** | **37.33 ± 4.51** | **All** |
|  | 13.36 ± 12.11 | 8 ± 0.63 | 37.50 ± 6.36 | bird1 |
|  | 37 | - | 37 | bird2 |
|  | 86.00 ± 14.72 | 88.00 ± 17.35 | - | bird3 |
|  | 23.50 ± 12.02 | 23.50 ± 12.02 | - | bird4 |
| **Mean bout duration ± SD [sec]** | **19.45 ± 20.35** | **19.33 ± 22.52** | **24.01 ± 3.76** | **All** |
|  | 7.06 ± 7.31 | 3.68 ± 0.28 | 21.84 ± 0.10 | bird1 |
|  | 28.35 | - | 28.35 | bird2 |
|  | 52.62 ± 11.51 | 52.32 ± 11.51 | - | bird3 |
|  | 16.83 ± 7.65 | 16.83 ± 7.65 | - | bird4 |
| **Mean call duration ± SD [sec]** | **0.508 ± 0.139** | **0.499 ± 0.127** | **0.495 ± 0.149** | **All** |
|  | 0.45 ± 0.14 | 0.45 ± 0.15 | 0.44 ± 0.11 | bird1 |
|  | 0.60 ± 0.16 | - | 0.60 ± 0.16 | bird2 |
|  | 0.51 ± 0.12 | 0.50 ± 0.10 | - | bird3 |
|  | 0.57 ± 0.18 | 0.57 ± 0.18 | - | bird4 |

**Table S2:** Summary statistics on male copulation calls for all, within-pair and extra-pair copulations (no separate column for the 4 instances without video). Note: bird1 performed within-pair and extra-pair copulations. Also note the difference between the number of calls bird 1 produced during intra-pair and extra-pair sexual behaviour.

# Supplementary Materials – References:

Haas F, Hansson B. 2008. Identification of 20 polymorphic microsatellite loci in European crow (*Corvus corone*) from existing passerine loci: PERMANENT GENETIC RESOURCES. Mol Ecol Resour. 8(4):846–850. doi:10.1111/j.1755-0998.2008.02087.x.

Tarr CL, Conant S, Fleischer RC. 1998. Founder events and variation at microsatellite loci in an insular passerine bird, the Laysan finch (*Telespiza cantans*). Mol Ecol. 7(6):719–731. doi:10.1046/j.1365-294x.1998.00385.x.

Wenzel MA, Webster LMI, Segelbacher G, Reid JM, Piertney SB. 2011. Isolation and characterisation of 17 microsatellite loci for the red-billed chough (*Pyrrhocorax pyrrhocorax*). Conserv Genet Resour. 3(4):737–740. doi:10.1007/s12686-011-9446-z.
